# Impact of Uric Acid Levels on Mortality and Cardiovascular Outcomes in Relation to Kidney Function

**DOI:** 10.3390/jcm14010020

**Published:** 2024-12-24

**Authors:** Young-Eun Kwon, Shin-Young Ahn, Gang-Jee Ko, Young-Joo Kwon, Ji-Eun Kim

**Affiliations:** 1 Department of Internal Medicine, Korea University Guro Hospital, Seoul 08308, Republic of Korea; eunkoda@naver.com (Y.-E.K.);; 2 Department of Internal Medicine, Korea University College of Medicine, Seoul 02841, Republic of Korea

**Keywords:** uric acid, kidney dysfunction, chronic kidney disease, mortality, cardiovascular

## Abstract

**Background**: Uric acid levels are linked to cardiovascular outcomes and mortality, especially in chronic kidney disease (CKD). However, their impact across varying kidney function remains unclear. **Methods**: We conducted a retrospective cohort study using the Observational Medical Outcomes Partnership Common Data Model (OMOP-CDM) database from a single center. Adult patients with at least one serum uric acid measurement between 2002 and 2021 were included and categorized by estimated glomerular filtration rate (eGFR): normal kidney function (≥90 mL/min/1.73 m^2^), mild dysfunction (60–89 mL/min/1.73 m^2^), moderate dysfunction (30–59 mL/min/1.73 m^2^), and advanced dysfunction (<30 mL/min/1.73 m^2^). The primary outcome was all-cause mortality with secondary outcomes being myocardial infarction (MI) and heart failure (HF). **Results**: A total of 242,793 participants were analyzed. Uric acid levels showed a U-shaped association with all-cause mortality in advanced kidney dysfunction, where both low (<3 mg/dL) and high (>10 mg/dL) levels increased mortality risk. In mild kidney dysfunction, lower uric acid levels were linked to better survival. HF risk increased linearly with higher uric acid, particularly in normal kidney function, while no significant association was found between uric acid and MI in any group. **Conclusions**: Uric acid levels are associated with mortality in a U-shaped pattern for advanced kidney dysfunction, while lower levels appear protective in mild dysfunction. These findings suggest the need for personalized uric acid management in CKD patients based on their kidney function.

## 1. Introduction

Uric acid is the end product of purine metabolism that has numerous deleterious effects including inhibition of nitric oxide production, endothelial dysfunction, and stimulation of the renin–angiotensin system, with further contributions to vascular smooth muscle cell growth, impaired arterial function, and increased oxidative stress [1]. In the general population, previous studies have revealed that elevated uric acid increases the risk of all-cause mortality and cardiovascular events such as ischemic stroke, myocardial infarction, heart failure, and hypertension [2,3,4].

Uric acid homeostasis is tightly controlled by the kidneys and intestines with the kidney assuming a pivotal role. Hyperuricemia is common in patients with chronic kidney disease (CKD), and it has been reported in 40–80% of patients with end-stage renal disease (ESRD) [5]. Nevertheless, the role of uric acids in CKD has long been controversial. Some studies showed CKD patients have a similar pattern of outcome as in the general population. Post hoc analysis of MDRD cohorts for CKD 3–4 patients reported that elevated uric acid increased the risk of mortality [6]. Another study with stages 3–5 CKD presented hyperuricemia as a risk factor for all-cause mortality and cardiovascular events [7]. Conversely, several studies in ESRD showed higher uric acid levels were associated with lower risk of all-cause and CV mortality [8]. Another study reported no association between hyperuricemia and all-cause and cardiovascular mortality in patients with CKD stage 3–5 [9].

However, previous studies had several limitations, such as including only a subset of the CKD population, analyzing risk according to arbitrarily defined uric acid categories, or including only a small number of participants. We therefore studied the effect of uric acid levels according to renal function on mortality and cardiovascular outcomes in a large cohort of subjects using Observational Medical Outcomes Partnership Common Data Model (OMOP-CDM).

## 2. Materials and Methods

### 2.1. Ethical Considerations

This study was approved by the institutional review board of the Korea University Guro Hospital (IRB No. 2022GR0455) and complied with the principles of the Declaration of Helsinki. Informed consent was waived by the board due to use of a de-identified clinical dataset. 

### 2.2. Study Design

This retrospective observational cohort study was based on data from the OMOP-CDM database at Korea University Guro Hospital. OMOP-CDM is a standard data schema that uses standardized terms for the electronic medical records [10]. The standardized clinical tables containing patient clinical data mapped to a unique concept identifier with de-identifying personal information were extracted and analyzed. 

The standardized tables for the study population from March 2002 to December 2021 were accessed for statistical analysis. Data of interest were queried and extracted using a structured query language (SQL) server. 

### 2.3. Study Population and Data Collection 

**Figure 1:**
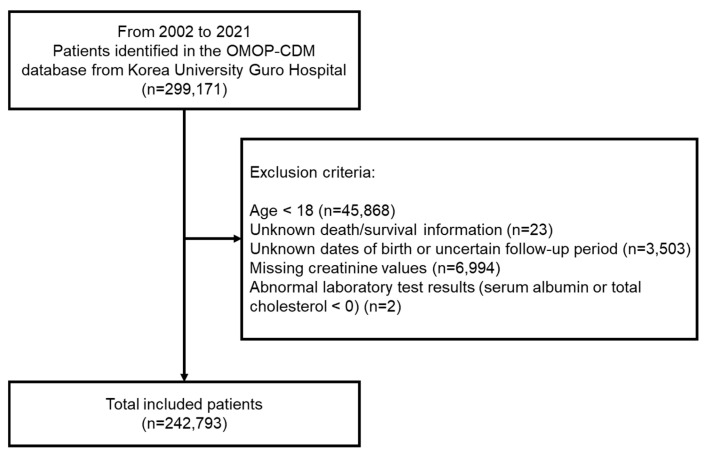
Flowchart of study participants selection process.

Adult patients with at least one measurement of serum uric acid levels during the study period were screened. Among the screened participants, the population with unknown death/survival information, unknown dates of birth, uncertain follow-up period, missing creatinine values and abnormal laboratory tests with serum albumin or total cholesterol levels less than zero were excluded (Figure 1).

We collected demographic and clinical data of study participants including age, sex, weight, height and comorbidities such as hypertension, diabetes, cancer and myocardial infarction at the time of uric acid measurement (defined as index date). The presence of comorbidity was defined by the issuance of an International Classification of Diseases-10 (ICD-10) code prior to the index date or by use of a related drug for hypertension and diabetes. We also collected the following laboratory data measured within 1 month from the index date: hemoglobin, serum albumin, serum creatinine, and total cholesterol. The estimated glomerular filtration rate (eGFR) was calculated using the creatinine-based CKD-EPI equation [11]. Prescription histories for uricosuric agents including allopurinol and febuxostat, treatment medications for acute gout attack including colchicine and steroid and statins were also collected. The use of each drug was defined as a prescription for more than 30 days within 1 year after the index date.

### 2.4. Study Groups

Participants were classified into groups according to their kidney function based on eGFR: normal kidney function (eGFR ≥ 90 mL/min/1.73 m^2^), mild kidney dysfunction (eGFR ≥ 60 and <90 mL/min/1.73 m^2^), moderate kidney dysfunction (eGFR ≥ 30 and <60 mL/min/1.73 m^2^), and advanced kidney dysfunction (eGFR < 30 mL/min/1.73 m^2^).

Participants were also stratified based on their serum uric acid levels measured on the index date. Since the serum uric acid level was a continuous variable, multiple groups were generated with uric acid level divided into 1 mg/dL unit, and the patients with a very low (<3 mg/dL) or very high (≥10 mg/dL) level of uric acids were classified into one group, respectively. For all analyses, those with 6–7 mg/dL of uric acid, which is generally considered a normal level, were classified as the reference group.

### 2.5. Study Outcomes

The primary outcome of this study was all-cause mortality. Secondary outcomes included the incidence of myocardial infarction requiring coronary intervention and the development of heart failure. The onset of myocardial infarction was defined by the ICD-10 code and the procedural code for coronary revascularization or thrombolysis. The development of heart failure was defined using the ICD-10 code and the procedure codes for echocardiography.

### 2.6. Mediation Analysis

We performed a mediation analysis to explore the potential mediating roles of eGFR and serum albumin in the relationship between uric acid levels and mortality. The mediation model specified uric acid as the independent variable (IV), 1-year mortality as the dependent variable (DV), and eGFR and albumin as mediators. The direct and indirect effects of uric acid on mortality were estimated with the total indirect effect comprising two pathways: one mediated by eGFR and the other by albumin. The model was constructed using Diagonally Weighted Least Squares (DWLS) as the estimator, which is appropriate for models with ordinal and non-normally distributed data. Model optimization was carried out using the NLMINB algorithm.

### 2.7. Statistical Analysis 

All statistical analyses, except for mediation analysis, were performed using STATA IC version 15.1. Data are expressed as means ± standard deviations for continuous variables and as n (%) for categorical variables. Cox regression analysis was conducted for estimating hazards for each outcome. The violation of the proportional hazards assumption was checked for all regression tests. For multivariable analysis, variables with a missing rate of 10% or more were discarded, and missing values were omitted for other variables. Therefore, multiple clinical variables including age, sex, hypertension, diabetes, cancer, myocardial infarction and laboratory tests including eGFR were used for multivariable analyses, while body mass index and social histories for smoking and alcohol consumption were not included as covariables. 

For the mediation analysis, the R package *lavaan* (version 0.6–17) was used to assess the mediating effects of eGFR and serum albumin on the relationship between uric acid levels and mortality. Bias-corrected bootstrapping with 5000 resamples was employed to test the statistical significance of the indirect effects. Model fit was evaluated using several indices, including the Comparative Fit Index (CFI), Tucker–Lewis Index (TLI), Root Mean Square Error of Approximation (RMSEA), and Standardized Root Mean Square Residual (SRMR). Standard errors for parameter estimates were obtained using the robust sandwich estimator. 

Cubic spline curves were plotted for representing the risk according to continuous variables. All tests were two-sided at a significance level of 0.05.

## 3. Results

### 3.1. Baseline Characteristics of the Participants

After screening and exclusion, we reviewed the OMOP-CDM data for a total of 242,793 participants. During 25.7 (4.1–77.4) months of follow-up, 11,317 (4.7%) participants died. The mean age of the total participants was 54.0 ± 16.2 years, and 50.5% were male. The mean blood uric acid level was 5.0 ± 1.8 mg/dL. 

According to kidney function, the participants were classified as normal kidney function (n = 191,127) and mild (n = 36,594), moderate (n = 9997), and advanced (n = 5075) kidney dysfunction groups, as mentioned in the Method Section. The advanced kidney dysfunction group showed a higher proportion of hypertension and diabetes compared to the other groups. Blood uric acid levels were 4.7 ± 1.5, 5.5 ± 1.7, 6.7 ± 2.3 and 7.8 ± 3.1 mg/dL in the normal, mild, moderate and advanced kidney dysfunction groups, respectively. The advanced kidney dysfunction group showed decreased hemoglobin, albumin and total cholesterol levels as well as increased uric acid levels compared to the other groups. The prescription rate for uricosuric agents, including allopurinol and febuxostat, were higher in the moderate or advanced kidney dysfunction groups compared to the normal and mild kidney dysfunction groups. Table 1 shows the detailed baseline characteristics of the total participants and participants included in each group.

### 3.2. All-Cause Mortality According to Blood Uric Acid Levels by Kidney Function

The association between baseline uric acid levels and all-cause mortality was assessed using multivariable Cox regression analysis. Participants were stratified per 1 mg/dL unit of their blood uric acid levels to analyze. When a uric acid level of 6–7 mg/dL was specified as the reference range, the all-cause mortality for all participants was decreased in the 3–4 and 4–5 mg/dL intervals and significantly increased in the 7–8 mg/dL and higher intervals. In particular, the participants with uric acid above 10 mg/dL showed a 2.3 times higher mortality risk compared to participants with a reference uric acid level (Table 2). 

**Figure 2:**
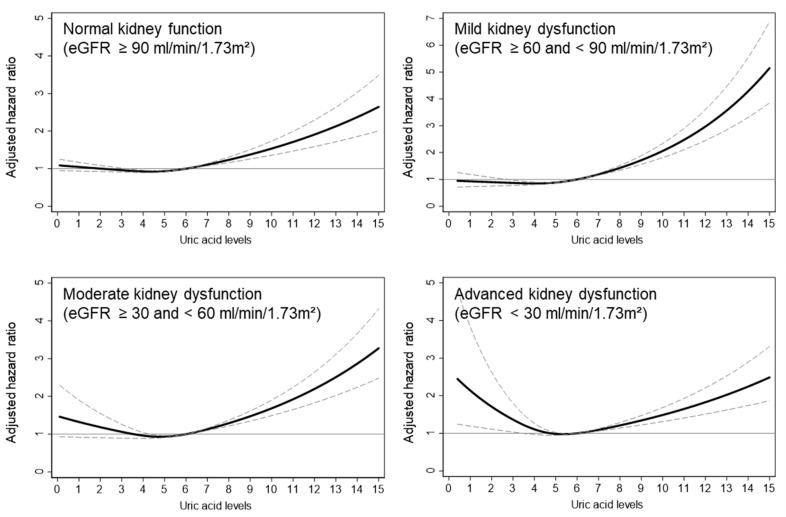
Adjusted hazard ratios for mortality according to uric acid levels by kidney function group. The figure shows adjusted cubic spline curves for the association between uric acid levels and all-cause mortality across four kidney function groups: normal kidney function, mild kidney dysfunction, moderate kidney dysfunction, and advanced kidney dysfunction. The solid black line represents the adjusted hazard ratios (HRs), while the dashed lines indicate the 95% confidence intervals. Adjustments were made for age, sex, hypertension, diabetes, cancer, myocardial infarction, body mass index, hemoglobin, blood urea nitrogen, creatinine, total cholesterol, and albumin. The reference uric acid level is 6–7 mg/dL.

We further examined the impact of uric acid levels on mortality within the different kidney function groups. In the normal kidney function group, the risk of mortality increased only when uric acid levels were elevated compared to the reference range. In contrast, the three kidney dysfunction groups showed different patterns on the spline graphs. In the mild kidney dysfunction group, there was a linear relationship with mortality risk decreasing at lower uric acid levels and increasing at higher levels. For the moderate kidney dysfunction group, similar to the normal kidney function group, low uric acid levels did not significantly impact mortality risk, but uric acid levels above the reference range were associated with an elevated risk. Severe hyperuricemia (uric acid ≥ 10 mg/dL) resulted in a 2.15-fold increase in mortality risk compared to the reference range. In the advanced kidney dysfunction group, both low and high uric acid levels, relative to the reference range, demonstrated a U-shaped relationship with increased mortality risk. Severe hyperuricemia (uric acid ≥ 10 mg/dL) and severe hypouricemia (uric acid < 3 mg/dL) were associated with 2.62-fold and 2.76-fold increased mortality risks, respectively. These cubic spline curves illustrating the relationship between uric acid levels and mortality risk are shown in Figure 2. 

### 3.3. Cardiovascular Risks According to Blood Uric Acid Levels by Renal Function

As secondary outcomes, we evaluated the risk of myocardial infarction and heart failure according to blood uric acid levels (Table 3). 

In total participants, myocardial infarction was developed in 3665 (1.5%) within 759 (124–2285) days after the index date. The 1-year incidence rate of myocardial infarction was 0.6, 1.9, 2.4 and 2.8% in the normal, mild, moderate and advanced kidney dysfunction groups, respectively. In multivariable Cox regression, the risk of myocardial infarction was not significantly different between blood uric acid levels in total participants as well as in each group. Supplementary Figure S1 illustrates adjusted hazard ratios for myocardial infarction according to uric acid levels stratified by kidney function group.

Heart failure was developed in 8890 (3.6%) within 740 (119–2247) days after the index date among the total participants. The 1-year incidence rate of heart failure was 1.0, 3.2, 5.7 and 6.2% in the normal, mild, moderate and advanced kidney dysfunction groups, respectively. Unlike the results regarding myocardial infarction risk, the risk of heart failure showed a significant linear association with blood uric acid levels among the total participants. That is, uric acid levels below 5 mg/dL and above 8 mg/dL showed decreased and increased risks of heart failure compared to the reference uric acid level, respectively. However, this linear pattern between heart failure risk and uric acid levels was sustained only in the normal kidney function group not in the other three groups. Supplementary Figure S2 depicts adjusted hazard ratios for heart failure.

### 3.4. Mediation Effect of eGFR and Albumin on the Uric Acid-Mortality Association 

**Figure 3:**
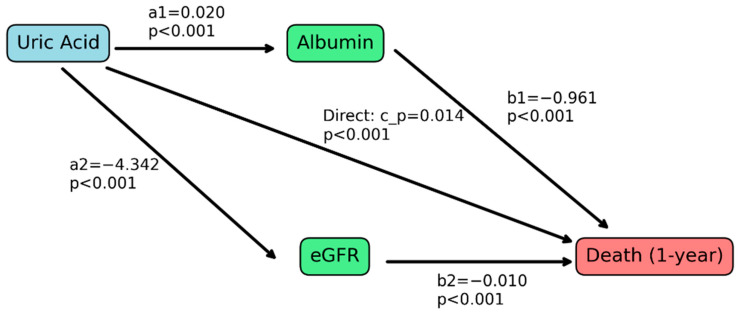
Mediation analysis of the association between uric acid levels and mortality. This figure illustrates the mediation pathways in the relationship between uric acid levels and 1-year mortality with the estimated glomerular filtration rate (eGFR) and serum albumin as mediators. Standardized coefficients for each path are displayed, and statistically significant mediation effects (*p* < 0.05) are indicated.

Based on the present result that the effect of uric acid was different by kidney function and the assumption about the role of uric acid as a nutritional marker, we conducted a mediation analysis of eGFR and albumin, as another nutritional marker, on the uric acid–mortality association (Figure 3). We found that both albumin and eGFR were statistically significant mediators of the uric acid–mortality association. And we found an indirect effect in which uric acid elevation, mediated by albumin, was related to a decreased risk of mortality (b = −0.019, *p* < 0.001), while there was another indirect effect in which uric acid elevation, mediated by eGFR, was related to an increased risk of mortality (b = 0.042, *p* < 0.001). The strength of the indirect effect was significantly higher in the eGFR-mediated model compared to the albumin-mediated model (b = −0.061, *p* < 0.001). Consequently, the total indirect effects through albumin and eGFR led to an overall increase in the risk of mortality with elevated uric acid levels. The direct effect of uric acid was also associated with an increased risk of mortality.

## 4. Discussion

In this large retrospective cohort study, we explored the relationship between uric acid levels and mortality, as well as cardiovascular outcomes, in patients stratified by kidney function. Our findings showed that elevated uric acid levels were linked to a higher risk of all-cause mortality particularly in patients with moderate and advanced kidney dysfunction. In advanced kidney dysfunction, both elevated and very low uric acid levels were linked to increased mortality, suggesting a complex relationship. Mediation analysis revealed that eGFR and serum albumin partially mediated the relationship between uric acid and mortality, highlighting the roles of kidney function and nutritional status in this association. Furthermore, uric acid levels showed a linear association with heart failure risk, especially in patients with normal kidney function, while no significant association was found with myocardial infarction risk.

The kidney plays a central role in uric acid excretion, and as kidney function declines, uric acid levels typically rise, making hyperuricemia common in CKD patients. Elevated uric acid has been associated with CKD progression, and it is linked to increased glomerular pressure, reduced blood flow, and the activation of inflammatory and vasoconstrictive pathways [12]. In addition to its association with CKD progression, uric acid has been widely studied as a biomarker in CKD due to its role in oxidative stress and endothelial dysfunction. While advanced biomarkers such as N-Gal, KIM-1, and non-coding RNAs show promise with greater sensitivity and specificity [13,14], uric acid remains a practical and easily accessible marker in clinical settings. However, the relationship between uric acid and hard outcomes, such as mortality, across different stages of CKD has been less thoroughly investigated.

Previous studies in patients with advanced kidney disease, including those on hemodialysis, have identified a J-shaped association between uric acid and all-cause mortality [15,16,17]. However, research on earlier stages of CKD is limited. The CRIC study reported a similar J-shaped mortality relationship in patients with eGFR between 20 and 70 mL/min/1.73 m^2^ [18], but few studies have explored the nuances of this relationship across various stages of kidney dysfunction. Our study expands on these findings by showing distinct patterns of uric acid and mortality association across different kidney function levels. In patients with advanced kidney dysfunction (eGFR < 30 mL/min/1.73 m^2^), we observed a U-shaped relationship, while in patients with mild kidney dysfunction, lower-than-expected uric acid levels were associated with better survival outcomes.

The U-shaped or J-shaped relationship in advanced kidney dysfunction could be explained by the dual role of uric acid. High uric acid levels contribute to endothelial dysfunction, oxidative stress, and inflammation, all of which increase mortality risk [12,15,19,20]. On the other hand, very low uric acid levels may reflect poor nutritional status, which is common in advanced CKD and is linked to protein-energy wasting (PEW). Since uric acid is influenced by dietary purine intake, low levels could indicate malnutrition. Malnutrition is often accompanied by increased inflammation and oxidative stress, both of which are associated with higher mortality in ESRD patients [21]. Our mediation analysis, which showed that albumin partially mediates the relationship between uric acid and mortality, supports this hypothesis. This suggests that low uric acid levels may not directly increase mortality but may serve as a marker of malnutrition, contributing to increased mortality risk. Additionally, uric acid’s potential role as an antioxidant could explain some of the direct effects we observed; the loss of uric acid’s free radical scavenging function in patients with very low levels may exacerbate oxidative stress [22,23,24]. While our study highlights the dual role of uric acid in advanced CKD, other factors such as volume overload, acid–base imbalance, and electrolyte disturbances may also interact with uric acid metabolism and contribute to mortality risk.

In contrast, lower uric acid levels were associated with improved outcomes in patients with mild kidney dysfunction, which was a pattern that was not observed in patients with normal kidney function. While the exact mechanisms remain unclear, one hypothesis is that in mild kidney dysfunction, lower uric acid levels reflect reduced metabolic stress or inflammation. Oxidative stress has been shown to increase even in early stages of CKD [25]. In these cases, lower uric acid may signal a reduction in these pathological processes, potentially leading to better outcomes. In patients with normal kidney function, metabolic balance is typically well maintained, which may explain why uric acid levels had less impact on mortality in this group. Another possible explanation is that while uric acid may affect mortality in CKD through its influence on oxidative stress and inflammation, other causes of death in individuals with normal kidney function could dilute the overall effect of uric acid on mortality. Since we did not assess specific causes of death in this study, further research is needed to clarify this hypothesis.

In addition to mortality outcomes, the effect of uric acid on cardiovascular disease has shown variability across studies. One study utilizing DOPPS data on dialysis patients found that higher uric acid levels were associated with lower cardiovascular risk [8]. However, other studies, including ours, have shown no association between uric acid levels and cardiovascular disease in patients with moderate or advanced kidney dysfunction [26,27,28]. In studies that included patients with normal or mild kidney dysfunction, one study reported that elevated uric acid increased the risk of composite cardiovascular events, including myocardial infarction and stroke [28]. In contrast, our study found no association with MI, while lower uric acid levels were linked to a reduced risk of heart failure. This discrepancy may be explained by the dual nature of uric acid—acting as an antioxidant at physiological levels but becoming a pro-oxidant at higher concentrations. The impact of uric acid on cardiovascular outcomes likely depends on the underlying health conditions and comorbidities of the study populations with different physiological contexts determining whether its antioxidant or pro-oxidant effects predominate.

While this study has several strengths, including the large cohort size and comprehensive analysis across different stages of kidney dysfunction, there are limitations to consider. The retrospective nature of the study limits our ability to infer causality, and the use of ICD-10 codes for defining comorbidities and outcomes may introduce some misclassification. Medical history was limited to diagnostic data for hypertension, diabetes, cancer, and myocardial infarction without detailed information on their severity or characteristics, which could impact the interpretation of their contributions to the observed associations. Additionally, we were unable to account for factors such as dietary habits, hydration status, and specific medications, such as diuretics, which can influence uric acid levels. Dehydration, which can elevate uric acid levels by reducing renal clearance, was not assessed in this study, leaving its potential impact on the observed associations uncertain. Moreover, while we explored the mediation effects of eGFR and serum albumin to better understand the relationship between uric acid levels and mortality, other unmeasured factors, such as inflammation, medication use, and liver function, may also play significant roles in this association. Additionally, renal incidents, such as acute kidney injury or CKD progression, were not included as secondary outcomes due to the limitations of the dataset. Future studies should consider incorporating renal outcomes to provide a more comprehensive understanding of the impact of uric acid levels on kidney disease progression. Finally, this study was conducted at a single center, which may limit the generalizability of our findings to broader populations. Future multi-center studies are warranted to validate these results and ensure their applicability across diverse patient populations.

## 5. Conclusions

In conclusion, this study demonstrates that the relationship between uric acid levels and mortality is highly dependent on kidney function. Elevated uric acid levels increase mortality risk, particularly in patients with moderate and advanced kidney dysfunction. However, in advanced kidney dysfunction, low uric acid levels are also associated with increased mortality, which is likely due to malnutrition and reduced antioxidant capacity. In contrast, lower uric acid levels appear to be protective in mild kidney dysfunction. These findings highlight the need for individualized management of uric acid levels in CKD patients with a focus on balancing the risks of both hyperuricemia and hypouricemia based on kidney function.

## Figures and Tables

**Table 1:** Baseline characteristics of study participants stratified by kidney function.

Variables	Total	Normal Kidney Function	Mild Kidney Dysfunction	Moderate Kidney Dysfunction	Advanced Kidney Dysfunction
	n = 242,793	n = 191,127	n = 36,594	n = 9997	n = 5075
Demographics					
Age, years	54.0 ± 16.2	50.6 ± 15.1	66.1 ± 14.2	69.9 ± 13.1	63.8 ± 15.1
Male sex, n (%)	122,647 (50.5%)	90,144 (47.2%)	24,518 (67.0%)	5177 (51.8%)	2808 (55.3%)
Body mass index, kg/m^2^	23.8 ± 4.0	23.8 ± 4.0	24.0 ± 3.6	24.2 ± 4.1	23.6 ± 4.0
Medical Histories					
Hypertension, n (%)	51,567 (21.2%)	33,835 (17.7%)	11,529 (31.5%)	4089 (40.9%)	2114 (41.7%)
Diabetes mellitus, n (%)	24,050 (9.9%)	15,471 (8.1%)	4828 (13.2%)	2343 (23.4%)	1408 (27.7%)
Cancer, n (%)	20,414 (8.4%)	15,312 (8.0%)	3674 (10.0%)	1101 (11.0%)	327 (6.4%)
Myocardial infarction, n (%)	2241 (0.9%)	1353 (0.7%)	631 (1.7%)	186 (1.9%)	71 (1.4%)
Laboratory Findings					
Uric acid, mg/dL	5.0 ± 1.8	4.7 ± 1.5	5.5 ± 1.7	6.7 ± 2.3	7.8 ± 3.1
Hemoglobin, g/dL	13.5 ± 1.9	13.6 ± 1.8	13.5 ± 2.0	12.0 ± 2.2	10.5 ± 2.3
Blood urea nitrogen, mg/dL	16.1 ± 9.3	14.1 ± 4.6	18.0 ± 6.4	27.8 ± 12.5	56.1 ± 25.8
Creatinine, mg/dL	0.9 ± 0.8	0.7 ± 0.2	1.0 ± 0.2	1.4 ± 0.3	5.1 ± 3.5
Estimated glomerular filtration rate, mL/min/1.73 m^2^	99.4 ± 22.2	108.1 ± 11.2	79.5 ± 8.7	48.0 ± 8.4	15.3 ± 8.2
Total cholesterol, mg/dL	179.0 ± 44.2	181.1 ± 42.9	175.2 ± 44.3	165.1 ± 53.5	153.5 ± 56.7
Albumin, g/dL	4.1 ± 0.5	4.2 ± 0.4	4.1 ± 0.5	3.8 ± 0.6	3.6 ± 0.6
Medication Prescription Histories (within 1 year)					
Allopurinol, n (%)	2659 (1.1%)	1108 (0.6%)	682 (1.9%)	527 (5.3%)	342 (6.7%)
Febuxostat, n (%)	2740 (1.1%)	932 (0.5%)	696 (1.9%)	694 (6.9%)	418 (8.2%)
Colchicine, n (%)	3176 (1.3%)	2257 (1.2%)	512 (1.4%)	278 (2.8%)	129 (2.5%)
Steroid, n (%)	34,710 (14.3%)	27,175 (14.2%)	5104 (13.9%)	1654 (16.5%)	777 (15.3%)
Statin, n (%)	47,000 (19.4%)	31,927 (16.7%)	9774 (26.7%)	3485 (34.9%)	1814 (35.7%)

**Table 2:** Hazard ratios for all-cause mortality associated with uric acid levels by kidney function group.

Uric Acid (mg/dL)	n	Unadjusted HR (95% CI)	*p*-Value	Adjusted HR * (95% CI)	*p*-Value
Normal kidney function					
<3	19,482	3.31 (2.99–3.66)	<0.001	1.03 (0.92–1.16)	0.604
3–4	44,280	1.42 (1.28–1.57)	<0.001	0.95 (0.85–1.06)	0.326
4–5	53,099	1.23 (1.11–1.36)	<0.001	0.97 (0.87–1.08)	0.529
5–6	38,214	1.10 (0.99–1.23)	0.075	1.01 (0.90–1.13)	0.901
6–7	21,534	1 (reference)		1 (reference)	
7–8	9037	1.10 (0.94–1.29)	0.229	1.22 (1.03–1.44)	0.022
8–9	3301	1.49 (1.21–1.84)	<0.001	1.77 (1.42–2.21)	<0.001
9–10	1233	1.95 (1.47–2.60)	<0.001	1.99 (1.47–2.68)	<0.001
≥10	947	2.43 (1.84–3.22)	<0.001	1.43 (1.06–1.93)	0.019
Mild kidney dysfunction					
<3	1784	1.96 (1.64–2.34)	<0.001	0.82 (0.68–0.99)	0.038
3–4	4465	1.30 (1.12–1.50)	<0.001	0.74 (0.63–0.86)	<0.001
4–5	7944	1.18 (1.04–1.35)	0.01	0.84 (0.73–0.96)	0.011
5–6	9140	0.95 (0.83–1.08)	0.421	0.83 (0.72–0.95)	0.006
6–7	6627	1 (reference)		1 (reference)	
7–8	3703	1.01 (0.86–1.19)	0.879	1.10 (0.93–1.30)	0.272
8–9	1724	1.42 (1.18–1.72)	<0.001	1.40 (1.15–1.71)	0.001
9–10	668	1.58 (1.21–2.07)	0.001	1.66 (1.25–2.19)	<0.001
≥10	539	3.24 (2.59–4.07)	<0.001	2.92 (2.29–3.71)	<0.001
Moderate kidney dysfunction					
<3	274	1.78 (1.31–2.43)	<0.001	1.25 (0.91–1.73)	0.166
3–4	578	1.12 (0.85–1.47)	0.421	1.00 (0.75–1.33)	0.992
4–5	1310	1.28 (1.05–1.56)	0.016	1.07 (0.87–1.33)	0.505
5–6	1907	1.02 (0.85–1.24)	0.805	1.02 (0.83–1.24)	0.859
6–7	1907	1 (reference)		1 (reference)	
7–8	1542	1.12 (0.92–1.37)	0.252	1.17 (0.95–1.44)	0.15
8–9	1095	1.41 (1.15–1.73)	0.001	1.49 (1.20–1.85)	<0.001
9–10	630	1.48 (1.16–1.88)	0.001	1.71 (1.32–2.20)	<0.001
≥10	754	2.57 (2.10–3.14)	<0.001	2.28 (1.84–2.82)	<0.001
Advanced kidney dysfunction					
<3	130	1.63 (0.73–3.65)	0.237	2.76 (1.21–6.30)	0.016
3–4	206	1.54 (3.86–2.76)	0.148	1.84 (1.00–3.39)	0.050
4–5	351	1.93 (4.20–3.11)	0.006	2.10 (1.28–3.45)	0.003
5–6	597	1.25 (5.79–1.98)	0.347	1.32 (0.83–2.12)	0.244
6–7	809	1 (reference)		1 (reference)	
7–8	769	1.59 (7.05–2.41)	0.029	1.80 (1.18–2.75)	0.007
8–9	671	1.57 (8.03–2.40)	0.038	1.75 (1.13–2.71)	0.013
9–10	534	1.78 (9.15–2.75)	0.009	2.08 (1.33–3.25)	0.001
≥10	1008	2.67 (10.85–3.86)	<0.001	2.62 (1.79–3.85)	0.000

* Hazard ratios (HR) and 95% confidence intervals (CI) are shown. Adjusted HRs are from multivariable Cox regression models with adjustments for demographic and clinical variables.

**Table 3:** Adjusted hazard ratios for myocardial infarction and heart failure according to uric acid levels by kidney function group.

Uric Acid (mg/dL)	n	Myocardial Infarction		Heart Failure	
		Adjusted HR * (95% CI)	*p*-Value	Adjusted HR (95% CI)	*p*-Value
Normal kidney function					
< 3	19,482	1.14 (0.80–1.62)	0.483	0.49 (0.38–0.62)	<0.001
3–4	44,280	1.05 (0.78–1.40)	0.757	0.57 (0.47–0.69)	<0.001
4–5	53,099	1.07 (0.83–1.39)	0.594	0.67 (0.56–0.81)	<0.001
5–6	38,214	1.20 (0.93–1.54)	0.152	0.83 (0.69–1.00)	0.046
6–7	21,534	1 (reference)		1 (reference)	
7–8	9037	1.14 (0.80–1.64)	0.462	1.05 (0.80–1.38)	0.722
8–9	3301	1.33 (0.78–2.27)	0.302	1.88 (1.35–2.61)	<0.001
9–10	1233	0.42 (0.10–1.70)	0.223	1.85 (1.15–2.99)	0.012
≥10	947	0.71 (0.22–2.30)	0.563	1.10 (0.56–2.15)	0.79
Mild kidney dysfunction					
<3	1784	0.92 (0.50–1.69)	0.793	0.84 (0.57–1.24)	0.379
3–4	4465	0.89 (0.59–1.35)	0.597	0.74 (0.55–1.00)	0.052
4–5	7944	0.90 (0.64–1.27)	0.566	0.84 (0.65–1.08)	0.171
5–6	9140	1.02 (0.75–1.40)	0.882	0.93 (0.73–1.18)	0.569
6–7	6627	1 (reference)		1 (reference)	
7–8	3703	0.96 (0.64–1.43)	0.832	1.33 (1.00–1.77)	0.051
8–9	1724	0.89 (0.52–1.53)	0.68	1.48 (1.04–2.11)	0.03
9–10	668	1.67 (0.93–3.00)	0.086	1.50 (0.90–2.50)	0.116
≥10	539	0.73 (0.26–2.08)	0.554	1.35 (0.74–2.49)	0.329
Moderate kidney dysfunction					
<3	274	0.57 (0.08–4.28)	0.584	0.64 (0.25–1.60)	0.339
3–4	578	0.69 (0.20–2.35)	0.554	1.00 (0.58–1.73)	0.999
4–5	1310	1.83 (0.98–3.42)	0.059	1.03 (0.68–1.56)	0.876
5–6	1907	1.39 (0.77–2.50)	0.27	1.40 (0.99–1.98)	0.055
6–7	1907	1 (reference)		1 (reference)	
7–8	1542	1.12 (0.59–2.11)	0.734	1.08 (0.73–1.59)	0.718
8–9	1095	1.33 (0.68–2.62)	0.406	1.47 (0.97–2.22)	0.067
9–10	630	1.93 (0.96–3.86)	0.064	1.65 (1.02–2.65)	0.04
≥10	754	1.83 (0.88–3.81)	0.106	1.69 (1.05–2.72)	0.031
Advanced kidney dysfunction					
<3	130	1.50 (0.43–5.23)	0.523	0.73 (0.22–2.42)	0.608
3–4	206	2.66 (1.12–6.32)	0.027	1.25 (0.57–2.76)	0.574
4–5	351	1.10 (0.43–2.83)	0.841	1.01 (0.51–2.04)	0.967
5–6	597	1.09 (0.51–2.32)	0.829	0.74 (0.40–1.39)	0.351
6–7	809	1 (reference)		1 (reference)	
7–8	769	0.93 (0.45–1.91)	0.837	0.93 (0.55–1.60)	0.801
8–9	671	0.98 (0.47–2.06)	0.966	1.19 (0.70–2.03)	0.519
9–10	534	0.77 (0.33–1.83)	0.555	1.06 (0.60–1.86)	0.853
≥10	1008	0.84 (0.39–1.78)	0.643	1.30 (0.79–2.14)	0.301

* Hazard ratios (HR) and 95% confidence intervals (CI) are shown. Adjusted HRs are from multivariable Cox regression models with adjustments for demographic and clinical variables.

## Data Availability

The data presented in this study are available on request from the corresponding author. Access to the data is restricted due to institutional policies and approval requirements.

## Supplementary Materials

The following supporting information can be downloaded at https://www.mdpi.com/article/10.3390/jcm14010020/s1, Figure S1: Adjusted hazard ratios for myocardial infarction according to uric acid levels stratified by kidney function group. The curves show adjusted hazard ratios (HRs) for myocardial infarction based on uric acid levels, stratified by kidney function groups. Adjustments were made for age, sex, hypertension, diabetes, cancer, myocardial infarction, body mass index, hemoglobin, blood urea nitrogen, creatinine, total cholesterol, and albumin. The reference uric acid level is 6–7 mg/dL. Dashed lines represent the 95% confidence intervals; Figure S2: Adjusted hazard ratios for heart failure according to uric acid levels stratified by kidney function group. The curves represent adjusted hazard ratios (HRs) for heart failure based on uric acid levels, stratified by kidney function groups. Adjustments were made for age, sex, hypertension, diabetes, cancer, myocardial infarction, body mass index, hemoglobin, blood urea nitrogen, creatinine, total cholesterol, and albumin. The reference uric acid level is 6–7 mg/dL. Dashed lines represent the 95% confidence intervals.
