# Supplementary material for: Impact of Uric Acid Levels on Mortality and Cardiovascular Outcomes in Relation to Kidney Function
Source: J Clin Med. 2024 Dec 24;14(1):20. doi: 10.3390/jcm14010020 (PMC11721403; doi:10.3390/jcm14010020)

Supplementary Figure 1. Adjusted hazard ratios for myocardial infarction according to uric acid levels stratified by kidney function group.

The curves show adjusted hazard ratios (HRs) for myocardial infarction based on uric acid levels, stratified by kidney function groups. Adjustments were made for age, sex, hypertension, diabetes, cancer, myocardial infarction, body mass index, hemoglobin, blood urea nitrogen, creatinine, total cholesterol, and albumin. The reference uric acid level is 6–7 mg/dL. Dashed lines represent the 95% confidence intervals.

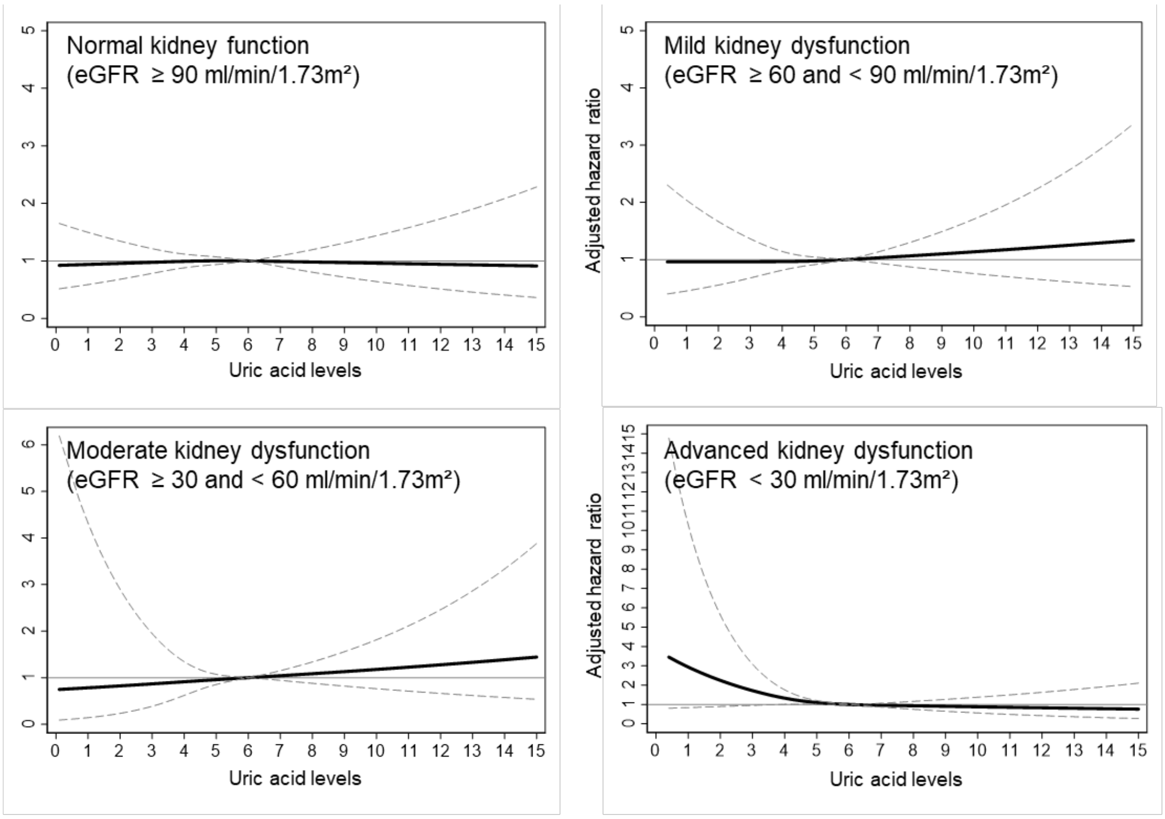

Supplementary Figure 2. Adjusted hazard ratios for heart failure according to uric acid levels stratified by kidney function group.

The curves represent adjusted hazard ratios (HRs) for heart failure based on uric acid levels, stratified by kidney function groups. Adjustments were made for age, sex, hypertension, diabetes, cancer, myocardial infarction, body mass index, hemoglobin, blood urea nitrogen, creatinine, total cholesterol, and albumin. The reference uric acid level is 6–7 mg/dL. Dashed lines represent the 95% confidence intervals.

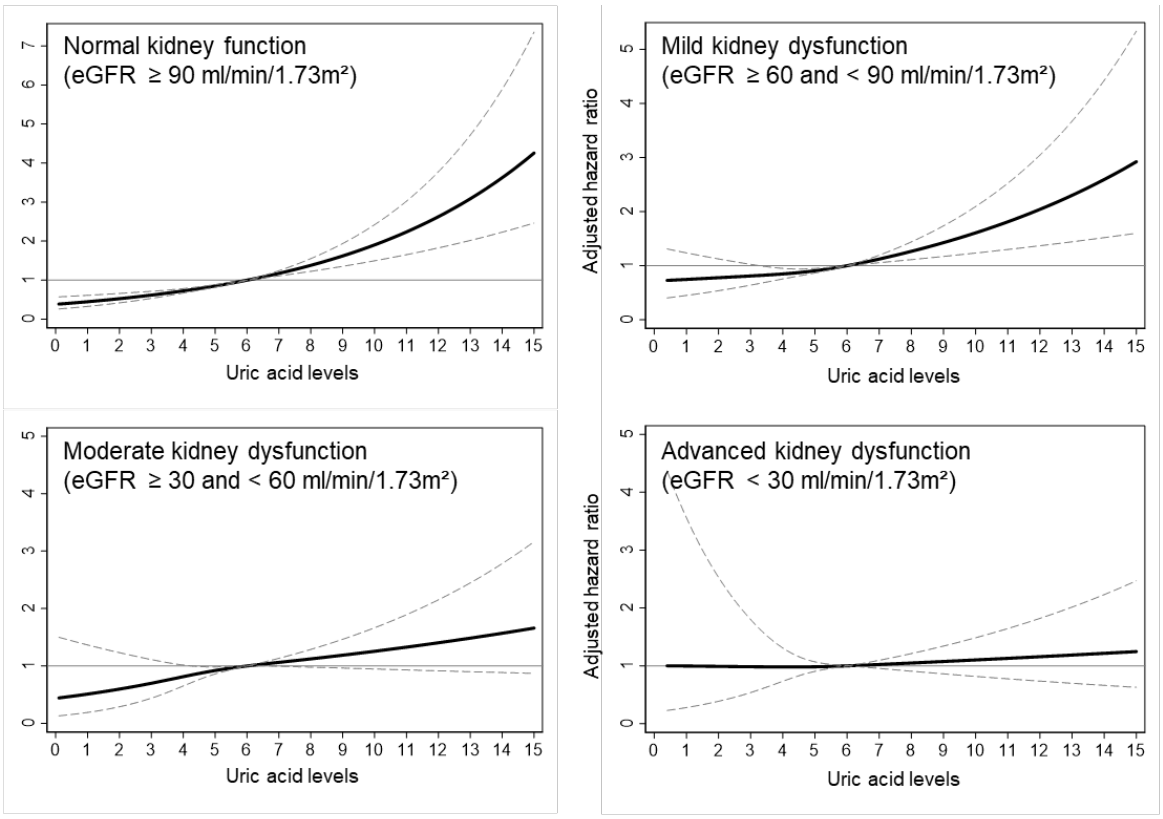

Supplement: Supplementary file 1 [file jcm-14-00020-s001.zip › jcm-3273990-supplementary.pdf]
